# Standards and Guidelines for Undergraduate Programs in Gerontology

**DOI:** 10.1093/geroni/igab046.2134

**Published:** 2021-12-17

**Authors:** Carrie Andreoletti, Laura Donorfio, Karen Kopera-Frye, Robert Maiden

**Affiliations:** 1 Central Connecticut State University, New Britain, Connecticut, United States; 2 University of Connecticut, Waterbury, Connecticut, United States; 3 New Mexico State University, Las Cruces, New Mexico, United States; 4 Alfred University, Alfred, New York, United States

## Abstract

Undergraduate programs (majors, minors, certificates) and continuing education programs in gerontology prepare students for entry-level careers in aging and increase competitiveness for graduate work in a variety of fields. Job growth in the field of gerontology is high, especially for positions requiring a bachelor’s degree and less. Gerontology education at this level is essential for meeting the growing demand for workers in social services and health services who understand the opportunities and challenges that come with increased longevity and global aging. This presentation will highlight the new recommendations for competency-based gerontology education for undergraduate and continuing education credentials outlined in the latest edition of AGHE Standards and Guidelines. Whether you are developing a new curriculum or revising an old one, we will offer suggestions for using the AGHE competencies and guidelines to ensure that your program adequately prepares students and offers them a competitive edge in today’s job market.

